# Resolution of obstructive sleep apnea after mandibular distraction osteogenesis in setting of delayed tongue–lip adhesion takedown: A case report

**DOI:** 10.1097/MD.0000000000012853

**Published:** 2018-10-19

**Authors:** Robyn S. Randall, Aaron Kian, Katherine Chin, Brooke French

**Affiliations:** a Keck School of Medicine, Los Angeles, CA; b University of Colorado School of Medicine; c Children's Hospital Colorado; d Department of Plastic Surgery, Children's Hospital Colorado, Aurora, CO.

**Keywords:** obstructive sleep apnea, Pierre Robin, tongue–lip adhesion

## Abstract

**Rationale:**

There is a high prevalence of obstructive sleep apnea (OSA) in patients with Pierre Robin sequence (PRS), and treatment approaches are highly variable. One approach is a temporary tongue-lip adhesion (TLA) that acts as a temporizing measure while the mandible continues to grow and is usually taken down at 1 year of age.

**Patient concerns:**

Side effects of prolonged tongue-lip adhesion and optimal workup and treatment of persistent OSA in the setting of a tongue-lip adhesion.

**Diagnoses:**

Pierre Robin sequence (PRS), persistent obstructive sleep apnea (OSA), and tongue-lip adhesion (TLA).

**Interventions:**

Mandibular distraction osteogenesis (MDO), adenotonsillectomy, and tongue-lip adhesion takedown.

**Outcomes:**

Resolution of OSA.

**Lessons:**

This case puts into question the efficacy of isolated TLA in infants with Pierre Robin sequence and OSA, and places emphasis on the importance of considering an earlier workup of other potential causes of obstruction and the potential need for MDO as a primary or adjunctive approach to treatment.

## 1 Introduction

Pierre Robin sequence (PRS) is a syndrome defined by micrognathia, glossoptosis, and upper airway compromise with or without cleft palate. It was first documented in 1923, and is estimated to occur in approximately 0.5 to 1.2 per 10,000 live births.^[2]^ The etiology is still unclear, but about one-third of PRS patients have associated genetic syndromes, most commonly Stickler syndrome and velocardiofacial syndrome. Hypoplasia of the mandible is thought to occur before the ninth week of development, forcing a posterior displacement of the tongue, which, in turn, can prevent closure of the palate leading to cleft palate.^[3,4]^ The most significant issues that present in infants with PRS are upper airway obstruction and feeding difficulties, which can lead to a failure to thrive and poor growth patterns that emerge over time, particularly before 10 months of age.^[5]^ Notably, there is a high prevalence of OSA in this population, impacting neurocognitive development as well.^[6,7]^ This highlights a need for early, effective, and definitive intervention.

Clinical signs to evaluate for upper airway obstruction in infants with PRS include stridor, labored breathing, diaphoresis, apnea, and restlessness. It is optimal for an experienced physician to assess for these over an adequate period of time while the patient is asleep, awake, or during feeding. An objective evaluation of respiratory compromise can include pulse oximetry, carbon dioxide retention, and polysomnography.^[5]^ However, although oximetry is specific for OSA, it is not sensitive for detecting airway obstruction.^[8]^ Thus, an increasing reliance on the apnea–hypopnea index (AHI) in sleep studies has been used to evaluate the overall success of airway management in these patients.^[9]^

Airway management in PRS patients ranges from minimally invasive such as prone or lateral positioning to surgical options such as tracheotomy. Prone/lateral positioning and close observation is the oldest established method and may be effective for PRS patients with only mild airway compromise. However, this method does not relieve airway obstruction for all infants, and it may limitation interaction with the parent from a prone position. Other options for conservative management include noninvasive respiratory support such as continuous positive airway pressure (CPAP) or noninvasive positive pressure, or a nasopharyngeal airway (trumpet). Traditional management of more severe airway compromise has been the tracheotomy. Glossopexy, or the tongue–lip adhesion (TLA), is associated with decreased morbidity and normalized weight gain, including an improvement in lowest oxygen saturation reading by 8.6% in one meta-analysis.^[10,11]^ It is estimated that airway issues that accompany PRS are expected to improve or resolve as the mandible continues to grow by the age of one, at which time palate repair is typically planned.^[1,15]^ However, this option has decreased in popularity due to its temporary effectiveness and the frequent need for nutritional support for >1 month and additional surgical procedures.^[12]^ Mandibular distraction osteogenesis is the most common surgical intervention, allowing patients with severe airway compromise to avoid a tracheotomy, and can provide a lasting modification. However, it is also one of the most invasive options.^[13]^

Timing and invasiveness in the treatment of upper airway obstruction in infants with PRS remains expansive and varied.^[1]^ We present a case report of delayed TLA takedown to highlight challenges in the approach to treatment. Ethical approval for this study was not necessary as this is a retroactive single case summary; however, informed consent was given for publication.

## 2 Case report

A 3-year-old boy with 9Q partial trisomy syndrome, PRS, obstructive sleep apnea (OSA), developmental delay, pulmonary hypertension, VSD, and G tube dependency presented to Craniofacial Clinic at Children's Hospital Colorado (CHC) for consideration of TLA takedown. The patient underwent TLA at 3 months of age at an outside hospital due to significant apnea and concern for upper airway obstruction unresponsive to prone or lateral positioning. A sleep study a year after the procedure showed mild improvement with a persistent apnea–hypopnea index of 4.3 events/h and a desaturation nadir to 83%. Due to swallow dysfunction, the patient was dependent on his G tube. On physical examination he had a sagittally short retrognathic mandible with the tongue in an anterior position secondary to his TLA.

A repeat sleep study interpreted by Otolaryngology and the Sleep team at CHC demonstrated severe sleep apnea with an apnea–hypopnea index of 31 and a nadir of 75%. Due to his severe apnea, TLA takedown at this time was considered a significant risk for worsening the condition. Nighttime oxygen was initiated; however, the patient did not tolerate CPAP. CT scan at age 3.1 years showed moderate micrognathia with slightly hypoplastic mandibular rami.

Bilateral mandibular osteotomies and distractor placement were initiated at age 3.6 years in an attempt to favorably modulate his OSA symptoms to facilitate TLA takedown. The patient was discharged on POD #4. Distraction was initiated after a latency period of 4 days. Initial X-rays obtained at 7 days demonstrated asymmetric diastasis between the sides. However, repeat X-rays 4 days later showed increased distraction on the right side compared with the previous film, so distraction was continued.

**Figure 1:**
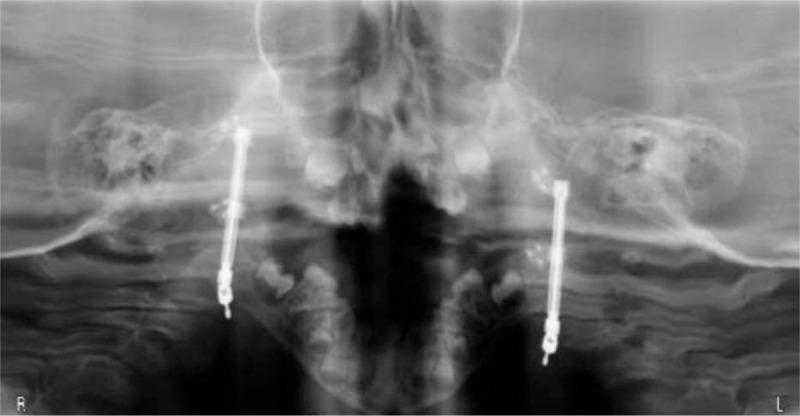
CT scan after 17 days of distraction. The superior portion of the right-sided distractor is in the glenoid fossa.

After 17 days of distraction, the patient presented for removal of external distraction arms. It was noted that he was unable to actively or passively close his mouth. CT scan showed the superior portion of the right-sided distractor in the glenoid fossa and anterior dislocation of the right mandibular condyle (Fig. 1). He was taken to the operating room for removal of the right-sided mandibular distractor and was discharged the next day. The proximal portion of the distractor had fractured a portion of cortical bone away with the screws and proximal distractor limb, likely due to incomplete/unfavorable osteotomy. An attempt at reosteotomy at that time was not undertaken due to loss of outer cortical table in desired area of replacement of the distractor.

Removal of the left mandibular distractor and repeat right mandibular osteotomy with placement of a right mandibular distractor were completed at the age of 3.9 years. Left-sided consolidation was confirmed to be complete during removal of the distractor. The distractor was turned BID for 17 days without complication.

Repeat sleep study at age 4 years, 1 month after cessation of distraction, showed continued moderate-to-severe OSA with an apnea–hypopnea index of 12.8 and mild snoring with mouth breathing. Sleep endoscopy was scheduled to evaluate for adenoid enlargement at the time of right distractor removal.

The right distractor was removed at age 4.3 years. Sleep endoscopy showed 75% adenoid obstruction that was very edematous, tonsils moderately encroaching on the airway, long soft palate and uvula, and severe right nasal obstruction due to septal deviation. Due to the high degree of obstruction, adenotonsillectomy was performed by otolaryngology at the time of distractor removal. Repeat sleep study 9 months later showed no OSA with an apnea–hypopnea index of 0.2 events/h. Given the resolution of OSA, TLA takedown was scheduled.

**Figure 2:**
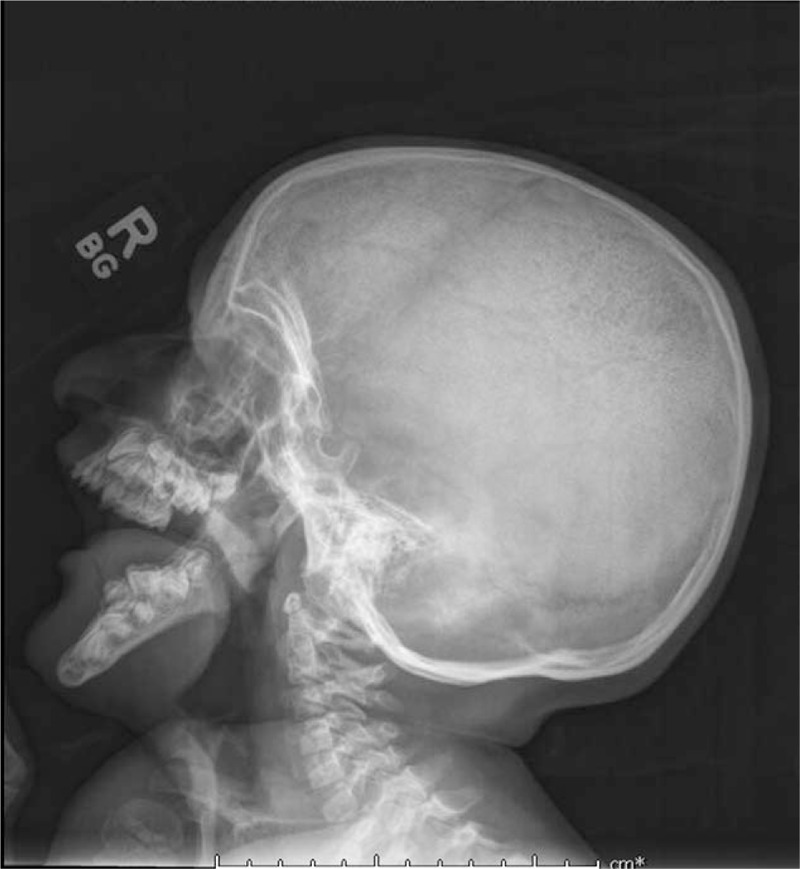
Lateral skull X-ray at age 5.

TLA takedown was performed at age 5.2 years. Previous CT scan at age 3.6 years and lateral skull X-ray at 5 years showed severely retroclined mandibular alveolus and teeth (Fig. 2). Dentistry was consulted intraoperatively and recommended consultation postoperatively with cleft palate team orthodontist for treatment of mandibular anterior ridge. On examination full complement of teeth was present in primary dentition and with no gross caries visible.

The TLA extended down to the anterior base of the tongue. Patient tolerated takedown well and was discharged on POD #2. Six days after TLA takedown the patient was admitted to the hospital due to coronavirus upper respiratory infection and difficulty managing his tongue and secretions. Otolaryngology was consulted and recommended glycopyrrolate for secretions and a course of dexamethasone. He maintained his airway throughout hospitalization without aggressive measures and was discharged after 3 days.

**Figure 3:**
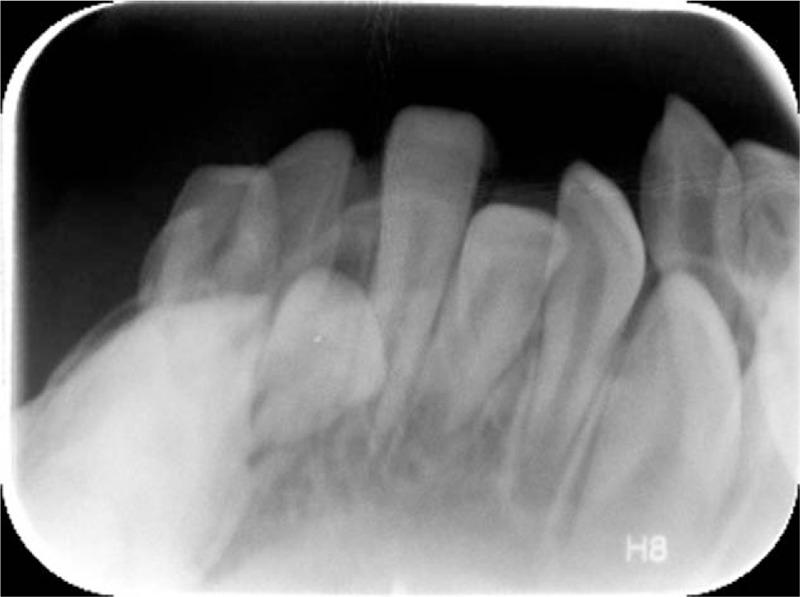
Mandibular occlusal radiograph from most recent appointment in 2018.

Four months later, per dentistry the patient's mandibular alveolus was better aligned but the teeth remained crowded and calculus was present. Repeat sleep study 5 months after surgery was essentially normal. At last follow-up, the patient has continued to do very well for the last 2.5 years (Fig. 3).

## 3 Discussion

TLA is a reliable temporizing measure to treat clinically significant airway obstruction in PRS patients.^[14]^ The primary indication for this patient's TLA was apnea, yet persistent OSA, was demonstrated on PSG. This is supported by a recent study of 18 subjects demonstrating that although TLA improved airway obstruction in PRS infants, its effect on *resolving* OSA is unpredictable.^[16]^ A recent meta-analysis of 90 patients reports that TLA reduced the mean apnea–hypopnea index from 30. 8 ± 22.3 to 15.4 ± 18.9, which is a 50% reduction.^[10]^ In addition, TLA improved the lowest oxygen saturation from 75.8 ± 6.8% to 84.4 ±7.3%. In this case it was not until the patient underwent MDO and adenotonsillectomy that the OSA resolved while the patient's TLA was still in place, suggesting early workup for adenotonsillar hypertrophy should be considered in cases with severe OSA.

In this case report, the patient retained his TLA until 5.2 years of age—far longer than anticipated for this temporizing intervention. Consequently, the patient's neuromuscular glossal and mandibular alveolar development were seriously impacted. Prolonged TLA resulted in a severely retroclined mandibular alveolus, embedded primary incisors in the floor of the mouth, and a steep dolichofacial mandibular growth pattern as measured by a gonial angle of 160° (Fig. 2). In addition, TLA and need to overcome apnea caused the patient's open mouth posture. The muscles of mastication were weak from disuse dystrophy because the patient's nutritional intake occurred through a gastronomy tube and from the inability to close the mouth and assume any tooth contact. The weakness of the masticatory muscles coupled with the unbalanced tension of the anterior digastric and masseter muscles at rest contributed to the extremely steep gonial angle resembling that of patients affected by cerebral palsy or muscular dystrophy.^[18,19]^ When the patient had returned for dental follow-up nearly 3 years after the TLA takedown, the position of the mandibular alveolus had significantly improved and three mandibular permanent incisors had erupted in a more upright fashion (Fig. 3). The TLA takedown contributed to a change in soft tissue equilibrium between tongue and lips whereby the force of the new resting tongue position within the mandibular arch could exert pressure on the lingual surfaces of the incisors and aid in improving the alveolar inclination. These changes can be explained by Moss's functional matrix theory which describes the influence soft tissue has on the growth of bone.^[20]^ There are no studies examining the impact of TLA on teeth, which is possibly due to the short time frame that TLA is typically present. However, future studies should examine if TLA has any adverse effects on occlusion and the development of teeth.

Mandibular distraction osteogenesis is a mandibular lengthening technique which is frequently used in the treatment of micrognathia in infants. It can lead to an improvement in respiratory status within a few days and has been shown to significantly decrease severity of OSA in appropriately selected patients. In addition, when compared with TLA, MDO achieves greater airway stability and a more rapid return to full feeding.^[1]^ However, failure rate is estimated between 2% and 9% and is higher in syndromic patients and those over 24 months of age.^[17]^ Indeed, our patient, who meets both of those higher risk criteria, experienced a unilateral distraction failure and delayed adenotonsillar evaluation.

The combination of the hypoplastic mandible and glossoptosis found in PRS predisposes this population to significant obstructive sleep apnea during early development. Although TLA prevents tongue retrusion, it does not address the anatomical obstruction from micrognathia, which is modified with MDO.

## 4 Conclusion

The treatment approach for upper airway obstruction found in patients with PRS remains highly variable, even within a single patient. Our patient was left with a surgically immobilized tongue until 5 years of age, and still experienced persistent OSA into early childhood that only resolved after MDO and adenotonsillectomy. This case puts into question the efficacy of isolated TLA in infants with PRS and OSA, and places emphasis on the importance of considering an earlier workup of other potential causes of obstruction and the potential need for MDO as a primary or adjunctive approach to treatment.

This case highlights the complicated nature of treating the PRS airway. Treatment continues to be highly individualized and dependent on a variety of factors, including severity of the airway, provider experience, and associated syndromes.

## Author contributions

**Conceptualization:** Brooke French.

**Data curation:** Robyn Randall, Aaron Kian.

**Formal analysis:** Robyn Randall.

**Project administration:** Brooke French.

**Supervision:** Brooke French.

**Writing – original draft:** Robyn Randall, Aaron Kian, Katherine Chin, Brooke French.

**Writing – review and editing:** Robyn Randall, Katherine Chin, Brooke French.

Robyn Randall orcid: 0000-0002-0473-5325.
